# Dual regulation of P-glycoprotein expression by Trichostatin A in cancer cell lines

**DOI:** 10.1186/1471-2199-13-25

**Published:** 2012-07-30

**Authors:** Trinidad Mata Balaguer, Angeles Gómez-Martínez, Pilar García-Morales, Javier Lacueva, Rafael Calpena, Lourdes Rocamora Reverte, Natividad Lopez Riquelme, Isabel Martinez-Lacaci, José A Ferragut, Miguel Saceda

**Affiliations:** 1 Fundación para la Investigación Biomédica del Hospital Universitario de Elche, Elche, Alicante, 03203, Spain; 2 Instituto de Biologia Molecular y Celular, Universidad Miguel Hernández, Elche, Alicante, 03202, Spain; 3 Unidad AECC de Investigación Traslacional en Cáncer, Hospital Universitario Virgen de la Arrixaca, Murcia, 30120, Spain

## Abstract

**Background:**

It has been reported that the histone deacetylase inhibitor (iHDAc) trichostatin A (TSA) induces an increase in MDR1 gene transcription (ABCB1). This result would compromise the use of iHDACs in combination with other cytotoxic agents that are substrates of P-glycoprotein (Pgp). It has also been reported the use of alternative promoters by the ABCB1 gene and the existence of a translational control of Pgp protein. Finally, the ABCB1 gene is located in a genetic locus with the nested gene RUNDC3B in the complementary DNA strand, raising the possibility that RUNDC3B expression could interfere with ABCB1 alternative promoter regulation.

**Methods:**

A combination of RT-PCR, real time RT-PCR, Western blot and drug accumulation assays by flow cytometry has been used in this study.

**Results:**

The iHDACs-induced increase in MDR1 mRNA levels is not followed by a subsequent increase in Pgp protein levels or activity in several pancreatic and colon carcinoma cell lines, suggesting a translational control of Pgp in these cell lines. In addition, the MDR1 mRNA produced in these cell lines is shorter in its 5^′^ end that the Pgp mRNA produced in cell lines expressing Pgp protein. The different size of the Pgp mRNA is due to the use of alternative promoters. We also demonstrate that these promoters are differentially regulated by TSA. The translational blockade of Pgp mRNA in the pancreatic carcinoma cell lines could be related to alterations in the 5^′^ end of the MDR1 mRNA in the Pgp protein expressing cell lines. In addition, we demonstrate that the ABCB1 nested gene RUNDC3B expression although upregulated by TSA is independent of the ABCB1 alternative promoter used.

**Conclusions:**

The results show that the increase in MDR1 mRNA expression after iHDACs treatment is clinically irrelevant since this mRNA does not render an active Pgp protein, at least in colon and pancreatic cancer cell lines. Furthermore, we demonstrate that TSA in fact, regulates differentially both ABCB1 promoters, downregulating the upstream promoter that is responsible for active P-glycoprotein expression. These results suggest that iHDACs such as TSA may in fact potentiate the effects of antitumour drugs that are substrates of Pgp. Finally, we also demonstrate that TSA upregulates RUNDC3B mRNA independently of the ABCB1 promoter in use.

## Background

Multidrug resistance (MDR) constitutes a major obstacle for success of cancer treatment. The MDR phenotype is responsible for resistance to a wide variety of anticancer drugs, such as anthracyclines, vinca-alkaloids and others [1]. Although several mechanisms could be involved in the acquisition of this phenotype, the role of two different membrane proteins, P-glycoprotein (Pgp) and multidrug resistance associated protein (MRP), has been well established [2-4]. Both proteins are members of the same ATP-binding cassette (ABC) superfamily of transport proteins. Pgp was first identified as a consequence of its overexpression in multidrug-resistant tumour cells, where it mediates the ATP-dependent efflux of a variety of chemotherapeutic agents. In addition to its role during the acquisition of the MDR phenotype, Pgp is expressed in normal tissues, both as a consequence of differentiation and also in response to environmental challenges, and it has been proposed to play a role as a cell protector against cellular toxins [5]. In addition, a general antiapoptotic role for Pgp has been proposed [6]. It is clear that Pgp has several functions in different cells and tissues.

Pgp is encoded by a multigene family in higher eukaryotes [7]. The ABCB1 gene (before MDR1) encodes the human Pgp. In cultured cells, constitutive overexpression of Pgp is mediated by changes in gene dosage or transcription. Pgp can also be transiently induced in cultured cells by a variety of stimuli, such as heat shock, UV radiation, and chemotherapeutic agents [8-11]. The regulation of Pgp expression has been mostly related to transcriptional control of the ABCB1 gene expression [8-11]. The proximal promoter of ABCB1 contains several regulatory regions, such as an inverted CCAAT box and a GC element, both of which are required for constitutive promoter activity in several cell lines [12-16]. It has been reported that in the colon carcinoma cell line SW620, the histone deacetylase inhibitor (iHDAC) trichostatin A (TSA), induces an increase in ABCB1 transcription through the inverted CCAAT box element, with the requirement of the NF-Y transcription factor [17]. This result can denote a big caveat, since a number of iHDACs are able to inhibit tumour growth, and several of them are used in clinical trials [18]. However, an increase in Pgp expression mediated by these inhibitors would hamper their combination with other cytotoxic agents that are substrates of Pgp. We have previously investigated in the human colon carcinoma cell lines SW620, HT-29 and HT-29/M6 the effect of TSA and Suberoylanilide Hydroxamic Acid (SAHA) on Pgp expression, demonstrating a translational control of Pgp expression [19]. The MDR1 mRNA produced in these cell lines is 285 bp shorter that the MDR1 mRNA produced in the human MCF-7/Adr and K562/Adr cell lines, both of them expressing Pgp protein. The different size of the MDR1 mRNA is due to the use of alternative promoters [19]. Interestingly, the ABCB1 gene is located in a genetic locus with the nested gene RUNDC3B in the complementary DNA strand. More specifically, several RUNDC3B exons are located in the complementary strand of the ABCB1 gene that corresponds with the intronic region between exon −1 and exon 1 of the MDR1 mRNA, raising the possibility of transcriptional interference between both genes. The study presented herein has been designed to gain insight in ABCB1 regulation determining whether the translational control of Pgp functions also in pancreatic cancer cell lines, the putative regulation of ABCB1 alternative promoters by iHDACs, and whether the expression of the ABCB1 nested gene RUNDC3B interferes with the expression of the MDR1 mRNA isoforms.

## Methods

### Cell lines and culture

IMIM-PC-1, IMIM-PC-2, RWP-1, PANC-1, Hs-766 T and BxPC-3 pancreatic carcinoma cell lines, as well as HT-29, HT-29/M6, SW-620, HTC-15, DLD-1, Colo 320 HSR (Colo-320) and LS-174 T human colon carcinoma cell lines, were kindly donated by the IMIM cell line repository (IMIM, Barcelona, Spain). MCF-7/Adr (adriamycin resistant subline derived from MCF-7) human breast carcinoma cell line was kindly donated by the Vincent T. Lombardi Cancer Center (Georgetown University, Washington D.C). Cells were grown at 37°C in 5% CO_2_, with DMEM (Dulbecco’s Modified Eagle Medium) (Gibco BRL Invitrogen, Carlsbad, CA) or RPMI (DLD-1 and Colo-320 cells) supplemented with 10% foetal calf serum (Biowhitaker, Velviers, Belgium), 2 mM L-glutamine (Gibco BRL Invitrogen, Carlsbad, CA), 1 mM sodium pyruvate when required (Gibco BRL Invitrogen, Carlsbad, CA), 50 U/ml penicillin and 50 μg/ml streptomycin (Gibco BRL Invitrogen, Carlsbad, CA). The murine leukaemia cell lines L1210, L1210R (daunomycin resistant subline derived from L1210), K-562 (human erythroleukaemia cell line) and K562/Adr (adriamycin resistant subline derived from K-562) were grown as previously described [20].

### Western blot

Treated or untreated cells were washed twice with PBS. After scraping the cells with PBS, they were centrifuged at 1000 x g for 5 minutes. L1210R and K562Adr cells which grow in suspension were washed twice with PBS. Pgp expression was determined by Western immunoblot using the monoclonal antibody (mAb) C-219 (Centocor Inc), as previously described [21,22], followed by enhanced chemoluminiscence (ECL) (Amersham International, Buckinghamshire, UK) to develop protein bands. Protein concentration in the cell lysates was determined by the Bradford method (Bio-Rad, Richmond, CA).

### Drug accumulation studies

Steady-state intracellular accumulation of the fluorescent substrate daunomycin (DNM) was determined as previously described, in the absence or in the presence of verapamil (VRP), an inhibitor of Pgp [23].

### Real time RT- PCR

To determine the level of MDR1 mRNA and RUNCD3B mRNA, total RNA from non treated or TSA-treated cells was isolated using the TRI reagent (Sigma-Aldrich, Co., St. Louis, MO). or the RNeasy Mini Kit (Qiagen GmbH, Germany). To eliminate potential DNA contamination, total RNA was treated with RQ1 DNase (Promega Corp. Madison, WI) for 30 min at 37°C followed by 2 min at 94°C. Reverse transcription of 1 μg RNA was performed using the TaqMan Reverse Transcription Reagents kit (Applied Biosystems Foster City, CA), according to the manufacturers’s instructions. Real time quantitative PCR was performed to amplify 20 ng of cDNA using the ABI PRISM 7700 Sequence Detector System (Applied Biosystems, Foster City, CA). Primers and probe to amplify MDR1 mRNA and RUNCD3B mRNA are commercially available (Applied Biosystems, Foster City, CA). Glyceraldehyde-3-phosphate-dehydrogenase (GAPDH) was used as endogenous reference in multiplex PCR. Primers and Taqman probe for this housekeeping gene are commercially available from Applied Biosystems (Taqman Pre-Developed Assay Reagents for gene expression). GAPDH Taqman probe was labeled with VIC in 5’ end as the reporter dye, and with TAMRA in 3’ end as the quencher dye. Relative expression of MDR1 and RUNCD3B mRNAs in tumour cell lines was determined by the comparative Ct method referred to the GAPDH housekeeping gene expression (ABI PRISM 7700 Sequence Detection System: User Bulletin #2, Applied Biosystems).

### RT-PCR analysis of the Pgp mRNA

To study the long 5^′^UTR MDR1 mRNA, total RNA from the different cell lines was isolated, treated with RQ1 DNase and reverse transcribed as described above. The cDNAs obtained were amplified by PCR using the appropriate set of primers. The primers’s sequences were: F1 (CATTCCTCCTGGAAATTCAACCT) and R1 (CTTCAAGATCCATTCCGACCTC) for MDR1 mRNA coming from ABCB1 upstream promoter (USP) and F2: GTTTCGCTATTCAAATTGGC and R2 (iso): CAGCCTATCTCCTGTCGCATT for MDR1 mRNA coming from both ABCB1 promoters (USP and DSP). The cDNAs were amplified by PCR as follows: 2 minutes at 94°C, and then 40 cycles of: 30 seconds at 94°C, 30 seconds at 55 to 61°C (depending on the set of primers), 45 seconds to 2 minutes (depending on the length of the amplified segment) at 72°C, and 7 minutes at 72°C. PCR products were resolved by electrophoresis on 0.5-2% (w/v) agarose gels. Bands were visualized with ethidium bromide.

## Results

### Effect of histone deacetylase inhibitors on Pgp mRNA expression in different pancreatic carcinoma cell lines

**Figure 1:**
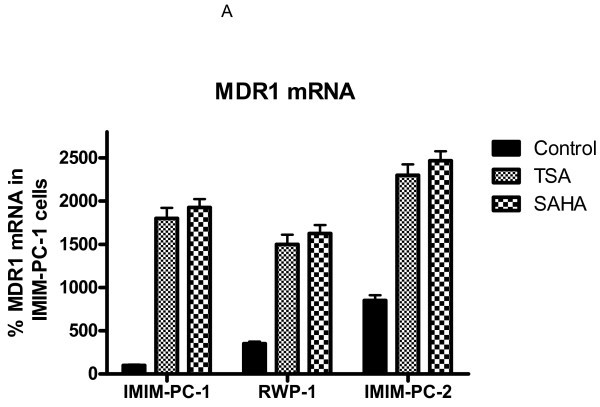
**TSA effect on Pgp levels and activity in pancreatic cancer cells.A**. MDR1 mRNA levels determined by real time RT-PCR. IMIM-PC-1, IMIM-PC-2 and RWP1 cell lines were treated or not with 1 μM TSA or 7.5 μM SAHA for 24 h. GAPDH mRNA was also determined as internal control. Results are shown as the mean ± SD of at least three independent experiments with three replicates in each experiment. **B**. Western blot analysis of Pgp expression in IMIM-PC-1, IMIM-PC-2 and RWP1 pancreatic carcinoma cell lines treated or not with 1 μM TSA for 24 h. As a positive control the Pgp expressing K-562/Adr cell line was included. β-Actin is included as internal control. **C**. Pgp activity in the pancreatic carcinoma cell lines IMIM-PC-1, IMIM-PC-2 and RWP1 treated or nontreated with 1 μM TSA for 24 h and in the presence or absence of 2.5 μM verapamil (Pgp inhibitor). Pgp activity was estimated as daunomycin accumulation determined by flow cytometry. Daunomycin accumulation was also determined in the L1210R cell line for positive control.

To determine iHDACs effects in MDR1 mRNA expression in human pancreatic carcinoma cell lines, we analyzed the level of MDR1 mRNA by real time RT-PCR in IMIM-PC-1, IMIM-PC-2 and RWP-1 human pancreatic carcinoma cell lines in the presence and absence of TSA and SAHA. Results in Figure 1A show that treatment with TSA or SAHA induced an increase in MDR1 mRNA levels in these cell lines. Despite this increase in MDR1 mRNA levels, we have previously reported that TSA and other iHDACs are able to inhibit cell growth and to induce apoptosis in these pancreatic carcinoma cell lines [24].

### Effect of histone deacetylase inhibitors on P-glycoprotein expression

To test whether the increase observed in the level of MDR1 mRNA correlates with an increase in Pgp protein, we analyzed Pgp protein levels by Western immunoblot in IMIM-PC-1, IMIM-PC-2 and RWP-1 cells in the presence or absence of TSA, using as a positive control the human erythroleukemia cell line K562Adr that expresses high levels of Pgp protein [20]. As shown in Figure 1B, there is no evidence of Pgp protein expression in these cell lines either before or after TSA treatment. To confirm that the lack of signal in the Western blot analysis of Pgp protein shown in Figure 1B was not due to the expression of a Pgp variant unable to react with the antibody used, Western blot analyses were performed with Pgp antibodies targeted againt different regions of the Pgp protein and we could not detect Pgp expression with any of the antibodies tested (data not shown). We also analyzed Pgp activity determining the accumulation of daunomycin (DNM), a fluorescent substrate of Pgp, in cells treated or untreated with iHDACs, using as a positive control L1210R cells. The presence of an active Pgp in these cell lines was demonstrated by the increased accumulation of DNM observed after addition of the Pgp inhibitor verapamil (VRP). Our results in Figure 1C show that no functional Pgp was present in IMIM-PC-1, IMIM-PC-2 and RWP-1 cell lines, either in the presence or in the absence of TSA.

### Translational control of Pgp mRNA in pancreatic carcinoma cell lines

**Figure 2:**
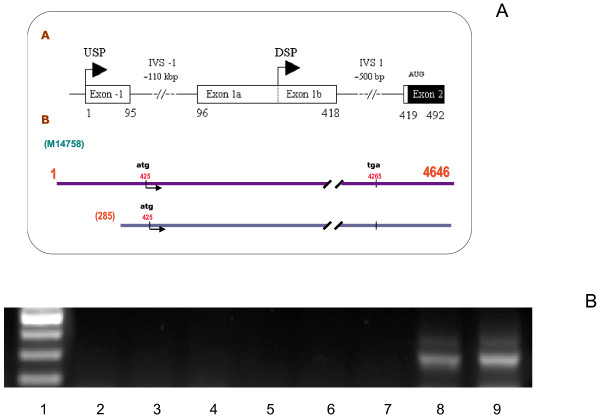
**Amplification by RT-PCR of the long 5**^**′**^**UTR MDR1 cDNA.A**. Diagram showing two ABCB1 promoters and the MDR1 mRNA isoform produced by each promoter. **B**. RT-PCR amplification with F1 and R1 primers of the long 5^′^ UTR of the MDR1 cDNA. Lane 1. Molecular markers.Lanes 2–7. IMIM-PC-1, IMIM-PC-2, RWP-1, Hs 766 T, BxPC-3 and PANC-1 cell lines. Lane 8.y 9, K-562/Adr and HTC-15 (positive controls).

**Figure 3:**
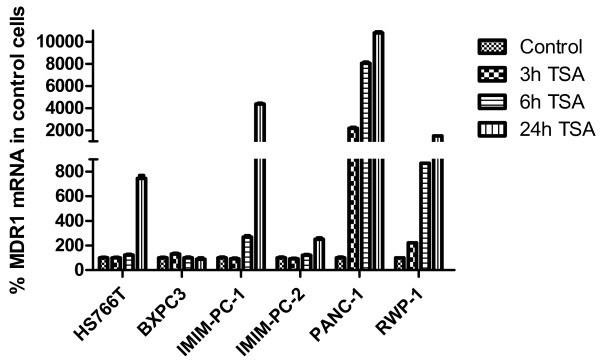
**TSA effects on MDR1 mRNA in different pancreatic carcinoma cell lines.** Hs-766 T, BxPC-3, IMIM-PC-1, IMIM-PC-2, PANC-1 and RWP1cell lines were treated for 0–24 hours with 1 μM TSA. As internal control GAPDH mRNA was also determined. Results are shown as the mean ± SD of at least three independent experiments with three replicates in each experiment.

After demonstrating that the increase in MDR1 mRNA levels due to iDHACs treatment did not correspond with an increase in Pgp protein expression or activity, and since these results resembled our previous findings in colon carcinoma cell lines [19], we decided to determine whether the MDR1 mRNA present in pancreatic carcinoma cell lines included the longer or the shorter 5^′^ UTR form (Figure 2A), and whether the translational control was a general phenomenon in pancreatic cancer cells. In order to do that, we extended this study to other pancreatic cell lines. Results in Figure 2B show that neither the pancreatic carcinoma cell lines, IMIM-PC-1, IMIM-PC-2 and RWP-1 nor the new added cell lines Hs 766 T, BxPC-3 and PANC-1 express the longer 5^′^UTR MDR1 mRNA, suggesting a generalized translational control of Pgp expression in human pancreatic carcinoma cell lines, since the new added cell lines were also negative for Pgp expression in Western blot analysis (data not shown). We have used K-562 ADR (erythroleukaemia cell line) and HTC-15 (colon carcinoma cell line) as positive controls, since both of them express a functional P-glycoprotein (19). Furthermore, Figure 3 shows the effects of TSA on MDR1 mRNA levels, as determined by real time RT-PCR in IMIM-PC-1, IMIM-PC-2, RWP-1, PANC-1, Hs 766 T and BxPC-3 cells. All the cell lines except BxPC-3 show an increase in MDR1 mRNA levels after TSA treatment.

### TSA regulates differentially the ABCB1 promoters

**Figure 4:**
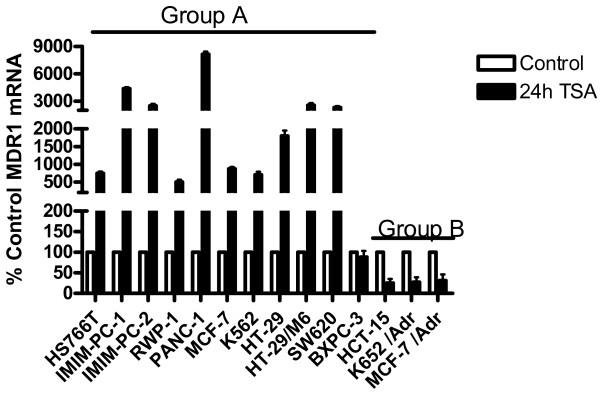
**Differential effect of TSA on ABCB1 alternative promoters.** Group A cell lines (Hs-766 T, IMIM-PC-1, IMIM-PC-2, RWP1, PANC-1, MCF-7, K-562, HT-29, HT-29/M6, SW620 and BxPC-3) expressing the short 5^′^UTR MDR1 mRNA and group B cell lines (HTC-15, K5-62/Adr and MCF-7/Adr) expressing the long 5^′^UTR MDR1 mRNAs were treated for 24 hours with 1 μM TSA. As internal control GAPDH mRNA was also determined. Results are shown as the mean ± SD of at least three independent experiments with three replicates in each experiment.

**Figure 5:**
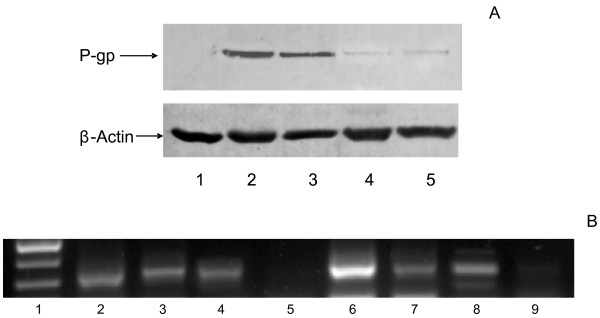
**TSA dual regulation of ABCB1 promoters.A**. Western blot analysis of Pgp expression in K-562 (lane 1), K-562/Adr (lane 2), negative and positive control respectively and the colon carcinoma cell lines Colo 320 (lane 3), DLD-1 (lane 4) and LS 174 T (lane 5). β-Actin is included as internal control. **B**. RT-PCR amplification with F1 and R1 primers of the long 5^′^ UTR of the MDR1 cDNA. Lane 1. Molecular markers. Lanes 2 and 3.K562/Adr and HTC-15 (positive controls). Lane 4, 6 and 8, Ls174T, Colo320 and DLD1 untreated cell lines. Lane 5, 7 and 9, Ls174T, Colo320 and DLD1 cell lines treated with 1 μM TSA for 24 h. **C**. Pgp activity in the erythroleukaemia cell lines K-562 (negative control), K-562 (d20) and K-562/Adr (positive control) treated or no treated with 1 μM TSA for 24 h and in the presence or absence of 2.5 μM verapamil (Pgp inhibitor). Pgp activity was estimated as daunomycin accumulation determined by flow cytometry. **D**. MDR1 mRNA levels determined by real time RT-PCR. K-562, K-562 (d450) and K-562/Adr cell lines were treated or not with 1 μM TSA for 24 h. GAPDH mRNA was also determined as internal control. Results are shown as the mean ± SD of at least three independent experiments with three replicates in each experiment.

The longer and the shorter 5^′^ UTR forms of MDR1 mRNA have been related to the existence of two ABCB1 alternative promoters [19,25]. To determine the effects of TSA on both ABCB1 gene promoters, we analyzed the levels of MDR1 mRNA after 24 h treatment with TSA in a panel of cell lines of different origin that have been divided in two groups. Group A includes cell lines that we have analyzed previously and use the downstream promoter (DSP) which transcribes the short 5^′^UTR MDR1mRNA (the pancreatic carcinoma cell lines described above, the colon carcinoma cell lines HT-29, HT-29/M6 and SW620, the MCF-7 breast carcinoma cell line and the K-562 erythroleukaemia cell line). Group B includes cell lines which use the upstream promoter (USP) that transcribes the long 5^′^UTR MDR1 mRNA (the HTC-15 colon carcinoma cell line, the MCF-7/Adr breast carcinoma cell line and the K-562Adr erythroleukaemia cell line). Results in Figure 4 clearly demonstrate differential effects of TSA on DSP and USP promoters and suggest that TSA is able to downregulate the USP promoter and upregulate the DSP promoter in the ABCB1 gene. To further demonstrate that TSA downregulates the USP promoter and therefore inhibits the expression of an active P-glycoprotein, we decided to include in this study three additional colon carcinoma cell lines: LS-174 T, Colo-320 and DLD-1 that express P-glycoprotein, as shown by Western blot analysis in Figure 5A. Figure 5B shows that these cell lines express the long 5^′^-UTR MDR1 mRNA and, furthermore, that TSA treatment decreased the levels of expression of the long 5^′^-UTR MDR1 mRNA in all of them. Even more, we used K-562 cell sublines resistant to different concentrations of daunomycin that have been generated in our laboratory by selective pressure with increasing concentrations of the drug. We have previously shown that resistance to daunomycin in this sublines was related to the active expression of P-glycoprotein, and that this expression correlates with the expression of the long 5^′^-UTR MDR1 mRNA [19]. As shown in Figure 5C, TSA was able to increase daunomycin accumulation in the K-562 d20 (resistant to 20 nM daunomycin), a subline that expresses P-glycoprotein and the long 5^′^-UTR MDR1 mRNA associated with the USP promoter [19]. This result suggests that TSA decreases P-glycoprotein expression leading to an increased accumulation of daunomycin in these cells. Futhermore, as shown in Figure 5C, TSA downregulated MDR1 mRNA, as determined by real time PCR in K-562 d450, a cell subline which expresses high levels of P-glycoprotein and also the long 5^′^-UTR MDR1 mRNA (19). Meanwhile, TSA increased the expression of the MDR1 mRNA in the parental K562 that does not express P-glycoprotein and expresses the short 5^′^-UTR MDR1 mRNA.

### TSA effect on RUNDC3B mRNA levels

**Figure 6:**
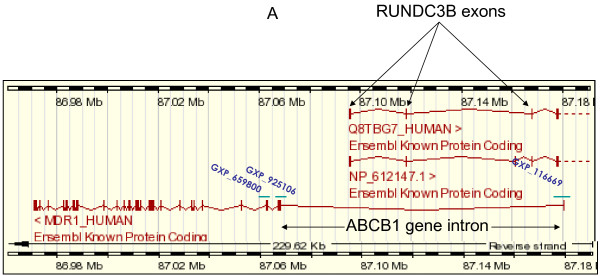
**Effect of TSA on RUNDC3B mRNA expression.A**. Diagram showing the overlapping of ABCB1 intron and RUNDC3B exons. **B**. Hs-766 T, BxPC-3, IMIM-PC-1, IMIM-PC-2, PANC-1, RWP1, HTC-15,MCF-7/Adr, LS 174 T, DLD-1 and Colo 320 cell lines were non treated or treated for 24 hours with 1 μM TSA and RUNDC3B mRNA expression were determined. As internal control GAPDH mRNA levels were also determined. Results are shown as the mean ± SD of at least three independent experiments with three replicates in each experiment.

The ABCB1 gene is located in a genetic locus with the nested gene RUNDC3B in the complementary DNA strand (see Figure 6A). Based on this special localization, we investigated whether the expression of RUNDC3B mRNA was regulated by TSA and whether the expression of this mRNA was related to the expression of the short or the long 5^′^UTR of the MDR1 mRNA. As shown in Figure 6B, TSA upregulates RUNDC3B mRNA expression levels independently of the MDR1 mRNA isoform (DSP or USP) expressed in the different cell lines.

## Discussion

In the present study, we demonstrate that the increase in MDR-1 mRNA levels induced by iHDACs inhibitors in pancreatic adenocarcinoma cell lines does not parallel an increase in Pgp protein or in Pgp activity. This observation is important since we and others have reported that the histone deacetylase inhibitors TSA and SAHA induce two major effects in several drug-resistant cell lines: down-regulation of Pgp and induction of apoptosis. In that sense, we have demonstrated that TSA markedly reduced Pgp expression in the L1210R drug-resistant murine cell line [20] and “sensitized” these cells to daunomycin [26,27], as shown in Figure 1C, where TSA treatment increases DNM accumulation in L1210R cells, suggesting that iHDACs might have a therapeutic potential against chemoresistant tumours. However, other authors had reported an increase in the steady-state level of MDR1 mRNA upon treatment with TSA [17], suggesting that iHDACs could not be utilized in combination with other cytotoxic agents that are substrates of Pgp. Our results in pancreatic carcinoma cell lines, which are in agreement with our previously published results in colon carcinoma cell lines [19], strongly suggest that expression of MDR1 mRNA is necessary but not sufficient for Pgp protein expression.

Next, we tried to identify the putative mechanisms involved in this phenomenon and we concluded that a translational blockade of Pgp expression takes place in pancreatic carcinoma cell lines, in agreement with our previous studies in colon carcinoma cell lines after TSA treatment [19] and in the human erytroleukaemia K-562 cell line [28].

Our data suggest that the translational blockade of MDR1 mRNA in the colon and pancreatic carcinoma cell lines and in K-562 cells could be overcome by alterations in the 5^′^ end of the MDR1 mRNA in the resistant variants of these cell lines. These results are especially relevant since we have previously demonstrated the relationship between the expression of the long 5^′^UTR MDR1 mRNA and the final expression of an active Pgp protein [19]. The origin and nature of these MDR1 mRNA isoforms became clear when Raguz et al. [25] reported the presence of an ABCB1 gene upstream promoter in breast carcinoma samples. Both promoters would translate the same protein because they use the same ATG codon, but the mRNA transcribed from the upstream promoter is approximately 285 bp longer in its 5^′^ end than the MDR1 mRNA transcribed from the downstream promoter. Our data, together with results published by other groups, strongly suggest that expression of MDR1 mRNA is necessary but not sufficient for Pgp protein expression, indicating that MDR1 mRNA is subjected to a negative translational control. During the acquisition of chemoresistance there is a switch from the downstream to the upstream ABCB1 gene promoter, and this promoter transcribes a MDR1 mRNA that is translated more efficiently. To sustain this hypothesis, we have demonstrated that the expression of an active Pgp protein correlates with the activation of the upstream promoter of the ABCB1 gene in several K-562 cellular sublines obtained by selective pressure with increasing concentrations of daunomycin [19] and Figure 5.

In addition, the results shown herein demonstrate that short and long 5^′^UTR MDR1 mRNAs are differentially regulated by the histone deacetylase inhibitor TSA, indicating that both promoters are differentially regulated by iHDACs. This is a compelling observation because TSA is able not only to downregulate the promoter responsible for active Pgp protein expression but also to induce apoptosis in colon and pancreatic carcinoma cells, sensitizing them to other chemotherapeutic agents that are substrates of Pgp. In addition, we observed that TSA increased MDR1 mRNA in the parental K-562 cells, whereas TSA decreased MDR1 mRNA levels in the daunomycin-resistant K-562 sublines which express Pgp protein and employ the USP promoter. This suggests again a differential regulation by TSA of both ABCB1 promoters.

We have also study the effect of TSA on the regulation of the RUNDC3B gene, since this is a nested gene transcribed from the ABCB1complementary DNA strand. These studies were designed to test the hypothesis that the regulation of the ABCB1 nested gene RUNDC3B could interfere with the alternative expression of both 5^′^UTR MDR1 mRNAs, since some of the exons of the RUNDC3B mRNA lie on the complementary strand of the ABCB1 gene, as shown in Figure 6A. Our results demonstrate that TSA is able to increase RUNDC3B mRNA levels, independently of the ABCB1 promoters that are active in these cell lines. RUNDC3B has been related to a more metastatic phenotype in breast cancer patients [29]. According to this, we could speculate a selection of RUNDC3B expression in colon and pancreatic carcinoma cell lines, instead of a functional Pgp protein expression, since expression of RUNDC3B could represent an additional advantage in their evolution to a more aggressive phenotype. However, taking into consideration our results, we cannot conclude that the expression of RUNDC3B could be in some way incompatible with the ABCB1 USP promoter expression forcing the cells to use the DSP promoter.

Although our data fit quite well with the existence of two ABCB1 promoters, some authors [30] have found that the additional 5^′^-UTR that is important for P-glycoprotein expression is due not to the use of an alternative promoter, but mostly to epigenetic changes in the region where this additional exon lies (112 kb upstream of the short MDR1 mRNA transcription initiation site). In fact, and based on chromatin immunoprecipitation assays and transient transfection of reporter genes under the control of the putative region where USP promoter lies, they concluded that the reason for the alternative 5^′^-UTR MDR1mRNA expression is due to epigenetic changes, mostly related to histone H3 acetylation. The situation in the putative upstream promoter cannot be explained only by histone acetylation because TSA generally produces a state of hyperacetylation due to histone deacetylases inhibition. Global general hyperacetylation is related to an increase in transcription and however upstream promoter transcription is inhibited. Epigenetic control of Pgp expression has been previously suggested by different authors, which found that demethylating agents were able to induce Pgp- mediated chemoresistance in different cellular models [31-33], We have some preliminary data with primary cultures derived from tumours of patients with colon cancer. In some of these cultures, we found that Pgp expression was lost. In some clonal populations obtained by extreme dilution of these cultures the long MDR1mRNA isoform and Pgp expression after treatment with DNA demethylating agents was recovered, suggesting that DNA methylation is involved in the expression of the MDR1mRNA isoforms (data not shown). However, it is unclear whether Pgp expression is regulated by two different promoters or by epigenetic mechanisms that modifies the activity of a single promoter. Since TSA is an epigenetic drug, from our results it is difficult to discriminate whether TSA downregulates the USP promoter or, alternatively modifies the epigenetic status of a specific region of the ABCB1 gene. In any case, we can affirm that TSA inhibits the expression of an active P-glycoprotein. The authors that argument the epigenetic changes as the main reason for the long 5^′^-UTR MDR1mRNA production suggest that the nested gene RUNDC3B is only expressed when the long 5^′^-UTR MDR1mRNA is expressed, or in other words that there is a correlation between the expression of both mRNAs. Our data do not support this hypothesis, because as seen in Figure 6B, RUNDC3B mRNA is expressed in cell lines that express the short 5^′^-UTR MDR1 mRNA, suggesting that the expression of both genes is not regulated simultaneously by the same epigenetic changes in a specific genomic region.

## Conclusions

The results shown herein show that the risk of a putative increase in Pgp expression after iHDACs treatment is clinically irrelevant since it does not render an active Pgp protein, at least in colon and pancreatic cancer cell lines. In addition, we have demonstrated that TSA regulates differentially both ABCB1 promoters and upregulates RUNDC3B mRNA expression levels independently of the ABCB1 promoter in use.

## Competing interests

The authors declare that they have no competing interests.

## Authors' contributions

Conceived and designed the experiments: MS, PGM and IML. Performed the experiments: TMB, AGM, LRR, PGM, NLR, JAF, MS. Contributed reagents/materials/analysis tools: JL, RC, JAF, IML. Wrote the manuscript: MS, PGM and IML. Critical comments: JL, RC, JAF. Approved the final version of the manuscript: MS.
